# Mapping seahorses in a Brazilian estuary: mangrove structures as key predictors for distribution and habitat preference

**DOI:** 10.7717/peerj.15730

**Published:** 2023-07-20

**Authors:** Anna Karolina Martins Borges, Rômulo Romeu Nóbrega Alves, Tacyana Pereira Ribeiro Oliveira

**Affiliations:** 1 Programa de Pós-Graduação em Etnobiologia e Conservação da Natureza, Universidade Federal Rural de Pernambuco, Recife, Pernambuco, Brazil; 2 LAPEC—Laboratório de Peixes e Conservação Marinha, Universidade Estadual da Paraíba, João Pessoa, Paraíba, Brazil; 3 Departamento de Biologia, Universidade Estadual da Paraíba, Campina Grande, Paraíba, Brazil; 4 Centro de Ciências Biológicas e Sociais Aplicadas, Universidade Estadual da Paraíba, João Pessoa, Paraíba, Brasil; 5 International Union for Conservation of Nature (IUCN) Species Survival Commission, Seahorse, Pipefish and Seadragon Specialist Group, Gland, Switzerland

**Keywords:** Syngnathids, Longsnout seahorse, Hippocampus reidi, Mangrove, Conservation

## Abstract

Planning for effective conservation demands an accurate understanding of the ecological aspects of species, particularly their distribution and habitat preferences. This is even more critical in the case of data-poor, rare, and threatened species, such as seahorses, mainly when they inhabit vulnerable ecosystems like estuaries. Given the importance of better understanding these parameters to design seahorse conservation strategies, we mapped the distribution and assessed habitat preferences of longsnout seahorses (*Hippocampus reidi)* in a mangrove estuary in a Brazilian protected area. Using generalised linear mixed-effects models we found that dense mangrove cover macro-habitats and shallow depths predicted seahorse sightings and higher densities. Furthermore, the selective index of micro-habitats used by seahorses showed that seahorses exhibited a preference for mangrove structures as holdfasts (*i.e.*, fallen branches). Due to the significant importance of mangroves in providing suitable habitats for *H. reidi* in estuaries, it is crucial to enforce the protection of these ecosystems in conservation and management strategies for the species.

## Introduction

Effective conservation requires careful systematic planning to ensure the correct application of resources and efforts (Fajardo et al., 2014). In turn, successful planning depends on a good understanding of species abundance, distribution, and habitat preferences to determine the area where conservation actions should be applied as a priority (Zhang & Vincent, 2018). Rare and threatened marine species comprise a challenge in this context, due to difficulties in assessing accurate bioecological data needed for determining the priorities and consequent conservation planning (Margules & Pressey, 2000; Stirling et al., 2016).

Seahorses (*Hippocampus* spp.) represent an important case study on this matter, as rare and threatened species with knowledge gaps. These iconic fish are known to inhabit threatened habitats (*e.g.*, estuaries, mangroves, coral reefs) and have peculiar life history characteristics, such as small home ranges, reduced mobility, pair-bonding, low reproductive rate, and long parental care (Foster & Vincent, 2004). These factors make seahorses particularly vulnerable to human impacts, especially considering habitat degradation and non-selective fishing (Vincent, Foster & Koldewey, 2011). Moreover, seahorses have been historically traded worldwide, primarily for traditional medicines and for the ornamental fish trade (Vincent, Foster & Koldewey, 2011). As a result of those pressures, of the 46 recognized species of seahorses, 14 are considered threatened at some level according to The International Union for Conservation of Nature’s Red List of Threatened Species (IUCN), and 17 are considered “Data Deficient”, (IUCN, 2022; Pollom et al., 2021). Moreover, the entire genus *Hippocampus* is listed in the Appendix II Convention on International Trade in Endangered Species of Wild Fauna and Flora (CITES, 2022). Those international listings demand imperative and pragmatic actions towards the protection and recovery of seahorse populations and habitats.

Seahorse species have different habitat dependency levels and preferences (Zhang & Vincent, 2018), and are generally found in shallow coastal habitats, including transitional ecosystems, such as estuaries (Bell et al., 2003; Claassens & Hodgson, 2018). Twelve species of seahorses have been recorded in estuaries (Harasti, Martin-Smith & Gladstone, 2012; Lourie et al., 2004; Perera, Dahanayaka & Udagedara, 2017; Rose et al., 2019; Yip et al., 2015), including one that is exclusively estuarine—*Hippocampus capensis* (Claassens & Harasti, 2020). Habitat degradation and loss are among the greatest threats to seahorses (Vincent, Foster & Koldewey, 2011) and the case of estuaries raises particular concern since they are considered one of the most imperiled marine ecosystems globally (Kennish, 2002). On this matter, the IUCN World Conservation Congress Resolution 95 establishes (among other measures) the need to focus on transitional habitats that are essential for syngnathid species (IUCN, 2020).

Despite providing crucial ecological functions such as breeding grounds, nurseries, and foraging areas for numerous species (Barbier et al., 2011; Gillanders et al., 2011; Vasconcelos et al., 2011), estuarine ecosystems are undergoing several alterations caused by coastal development, fishing pressure, pollution, climate change, and invasive species (Aylesworth et al., 2015; Barletta & Lima, 2019). Tropical estuaries are often associated with another threatened ecosystem, mangroves, which provide approximately $1.6 billion a year in ecosystem services (Godoy, Meireles & Lacerda, 2018; Primavera et al., 2019). Brazil has a prominent position in the distribution of mangrove forests, holding the second-largest extension of mangroves on the planet (939,685 ha; Diniz et al., 2019; Gomes, Vescovi & Bernardino, 2021). All mangroves in Brazil are considered Permanent Preservation Areas (PPAs) (Brasil, 2012) and were targeted by the National Action Plan for Mangroves (Plano de Ação Nacional para a Conservação das Espécies Ameaçadas e de Importância Socioeconômica do Ecossistema Manguezal PAN Manguezal; Ministry of Environment and Climate Change of Brazil (MMA), 2019), which aimed at improving the conservation status of mangrove ecosystems, reducing degradation and protecting the focal species that inhabit these environments. However, mangroves are highly vulnerable and considered the least protected coastal ecosystem by Brazilian integral protection areas mainly due to the lack of inspection for compliance with legislation (Ferreira & Lacerda, 2016; Vilar, Joyeux & Spach, 2017).

Mangrove estuaries comprise a key habitat for the longsnout seahorse *Hippocampus reidi* Ginsburg, 1933 in Brazil. The species is distributed from Cape Hatteras, United States, to the Gulf of Mexico and Brazil (Lourie, Pollom & Foster, 2016). Its occurrence is reported along all the Brazilian coast, from the northern state of Pará to the southern state of Rio Grande do Sul (Rosa, 2005; Chao et al., 1982), and can be found in shallow reefs, rocky shores, and seagrass beds (Dias-Neto, 2011; Rosa et al., 2005; Rosa et al., 2007; Oliveira, Castro & Rosa, 2010; Freret-Meurer et al., 2018). However, *H. reidi* occurrence has been mostly reported in mangrove estuaries on the NE coast (Rosa et al., 2007), using structures such as mangrove roots, fallen branches, macroalgae, and sponges as micro-habitats (Dias & Rosa, 2003; Rosa et al., 2005; Rosa et al., 2007; Mai & Rosa, 2009). Indeed, Aylesworth et al. (2015) reported the presence of mangrove structures as habitat predictors of *H. reidi* occurrence, besides calm, shallow estuarine waters, and warm temperatures in NE Brazil. Nonetheless, little is known about the factors that influence seahorse distribution and habitat preferences in estuarine environments (Aylesworth et al., 2015).

*Hippocampus reidi* is classified as “vulnerable” in the Brazilian national red list of threatened species (Ministry of Environment and Climate Change of Brazil (MMA), 2022) and is categorized as globally “near threatened” by the IUCN (Oliveira & Pollom, 2017). As a threatened and data-poor species, knowing distribution patterns and habitat use in key estuarine environments is crucial to analyze the species’ threats and guide appropriate actions for its conservation. Therefore, in this study, we sought to (i) map the distribution of *H. reidi* in a mangrove estuary located in a marine protected area (MPA) in NE Brazil; and (ii) explore the effects of environmental and habitat characteristics on the distribution and density of *H. reidi*. Hence, we aim to contribute to filling gaps in knowledge about *H. reidi* and the distribution of seahorses in estuarine and mangrove habitats, thus contributing to strengthening strategies towards such crucial ecosystems for seahorse conservation.

## Materials & Methods

### Study area

**Figure 1:**
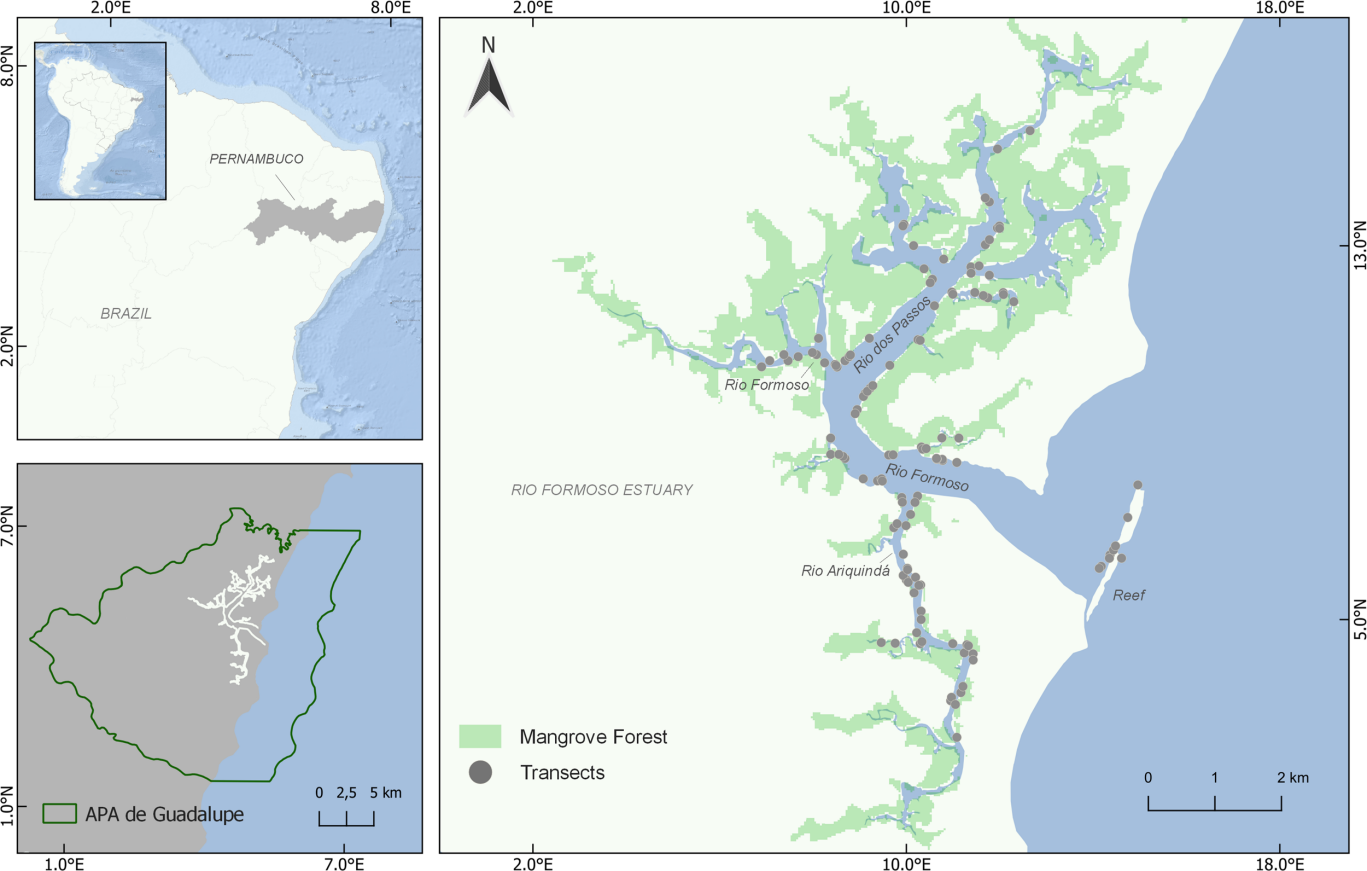
Location of the Rio Formoso Estuary (Pernambuco State, Brazil) and sampling sites (transects) for mapping seahorse distribution. Mangrove distribution and coverage are based on 2021 data from MapBiomas (available in https://mapbiomas.org/).

The study was carried out in the Rio Formoso Estuary (RFE), south coast of Pernambuco state, northeastern Brazil (8°41′14″S, 35°05′48″W; Fig. 1). The RFE has an area of approximately 2,724 hectares, comprised of three main rivers (Rio Formoso, Rio dos Passos, and Rio Ariquindá) (CPRH, 2011; Lira, Zapata & Fonseca, 1979). The estuary margins are covered by extensive mangrove forests formed by three mangrove tree species(*Rhizophora mangle* Linnaeus, *Laguncularia racemosa* Gaertn. and *Avicennia schaueriana* Staf. and Leechman) (Silva et al., 2003). Tourism, artisanal fishing, and crab and shellfish collection are the main economic activities in the estuary (Silva et al., 2003; Silva et al., 2009).

The RFE is located in the Guadalupe Environmental Protection Area (*Área de Proteção Ambiental de Guadalupe—APAG*), created with the objective of mitigating socio-environmental conflicts, which mostly involve disordered tourism, predatory fishing, shrimp farming, and agricultural (sugar cane) pollution (Santos, 2002; Araújo, Alves & Simões, 2014). Previous studies have recorded the occurrence of *H. reidi* in the Rio Formoso Estuary (Rosa et al., 2007; Oliveira, Castro & Rosa, 2010; Aylesworth et al., 2015), and recently a decline of 60–90% of the seahorse population in the Ariquindá River has been verified (TPR Oliveira, 2019, unpublished data).

### Sampling

We conducted visual surveys through snorkeling (Aylesworth et al., 2015; Woodall et al., 2018) to assess the distribution and density of *H. reidi* in the RFE using random linear transects (50 × 2 m). We conducted 125 transect surveys throughout the estuary from December 2020 to July 2021 (except March and April), surveying a total of 12,500 m^2^ (Fig. 1). To ensure a systematic sampling approach, we used a random-stratified approach (Miller & Ambrose, 2000), dividing the RFE area into 53 sample zones of 500 m in length and performing at least one transect survey at each zone. The location, direction, and number of transects in each sampling zone were haphazardly chosen, mainly considering the habitat availability for seahorses, *i.e.,* sand beaches without essential habitat availability and areas where the occurrence of seahorses is unlikely due to low salinity were excluded. To minimize possible effects of seasonality, we sampled all rivers every month, performing transects at all portions of the estuary (lower, middle, and upper), in both the dry and rainy seasons. None of the transects were sampled more than once, and a minimum distance of 50 m between the transects was considered. In addition to the estuarine-mangrove areas, the surveys were also carried out in a reef environment in the mouth of the estuary (Fig. 1). Surveys were always performed during similar tide levels (0.4–0.6 m) and with a minimum visibility of 50 cm.

For each transect, we sampled the following abiotic variables: salinity (10–37; mean: 30 ± 6.9), surface water temperature (26.5–33.8 °C; 29.4 ± 1.5 °C), visibility (using a Secchi’s disc: 0.15–1.50 m; 0.44 ± 0.24 m), and local depth (0.16–1.50 m; 0.48 ± 0.25 m). We also visually described and categorized the predominant type of macro-habitat in each transect (Table 1; adapted from Claassens, 2016). These macro-habitats differ primarily in terms of bottom type and presence and extent of mangrove coverage. Once a seahorse was sighted, we determined the geographic position (recorded with a GPS device), sight depth, and holdfast used by the seahorse. We also estimated the availability of micro-habitat from the percentage of benthic cover measured with a square of 0.25m^2^ centred around each seahorse found (adapted from Correia et al., 2018). When present, algae were classified according to the “Reef Check Brasil” monitoring manual (Ferreira et al., 2018). All information was collected without removing the seahorses from the water and following animal ethical protocols (SISBIO license no. 59294-2).

**Table 1:** Macro-habitat categories of the sampled transects in the Rio Formoso Estuary. Data are as follows: number of transects performed (total transects), number of transects in which seahorses were sighted (seahorse transects), and average density of seahorses found in each type of macro-habitat. Density values are given as mean ± confidence interval (range).

**Macro-habitat type**	**Total transects (n)**	**Seahorse transects** **(n)**	**Density (** **ind./m^2^)**
**I—Dense mangrove**: characterized by the presence of a dense mangrove on the banks of the river, with a large presence of submersed roots, leaves, and fallen branches of mangrove trees, mainly *Rhizophora mangle*, which contributes to the formation of complex habitats favoring the presence of secondary habitat components, such as sponges, algae, oysters, and octocorals.	73	48	0.032 ± 0.011 (0–0.34)
**II—Sparse and/or tidal flat mangrove:** mangroves present on the banks of the river with more sparse distribution, with the presence of clearings and, consequently, lower availability of submersed roots. In these places, tidal flats can also form during the lower tides, with the roots being totally or partially uncovered. They may also contain the secondary habitat components associated with the mangrove roots mentioned for type I habitat but in smaller quantities.	23	13	0.019 ± 0.011 (0–0.1)
**III—Reef environment**: formed mainly by rocky (inorganic) reefs with algae, coral, and sponge coverage.	9	0	0
**IV—Algae and sponge banks:** banks formed by agglomerates of different species of algae or sponges or, in some cases, with the presence of both.	3	2	0.006 ± 0.005 (0–0.0)
**V—Sandy substrate with rocks:** environment composed of sandy bottom and sparsely distributed rocks, which might contain algae or sponge coverage.	17	2	0.001 ± 0.001 (0-0.01)

### Data analysis and mapping procedures

All density means are reported with a 95% confidence interval. Seahorse density was compared among the three rivers and among the different macro-habitat types by the Kruskal-Wallis test (*p*-values were adjusted for multiple comparisons with Bonferroni correction when differences were observed). We used the Chi-squared test with Yates correction to compare holdfasts used by seahorses in each river. Mangrove coverage was extracted from the MapBiomas Platform (http://mapbiomas.org, accessed November 2021) for maps. We used a Kernel Density tool in the QGIS software program (https://qgis.org/) to determine hotspots of seahorse density.

A model approach was adopted to explore the effect of environmental variables on seahorse distribution. We built models considering two different response variables: (1) data on the presence/absence of seahorses; and (2) seahorse density. We used ‘generalized linear mixed-effect models’ (GLMMs) *via* the ‘glmmTMB’ function in the ‘glmmTMB’ package (Brooks et al., 2022). A negative binomial distribution was used to model the response variable based on presence/absence data and a generalized Poisson distribution was chosen for the model based on density data. Salinity, temperature, depth, and type of macro-habitat (Table 1) were included as fixed effects and the river where each transect was performed was included as a random effect for both models. The variables were primarily used in two global models and the ‘dredge’ function within the ‘MuMIn’ package (Bartón, 2019) was subsequently used to perform model selection and derive the optimal set of predictors for both models. This function fits different models comprising all combinations of the fixed effects and ranks them by the corrected Akaike information criterion (AICc). Next, the ‘DHARMa’ package (Hartig, 2019) was used to assess the dispersion of model residuals and to identify possible over/underdispersion or zero inflation to validate the model assumptions. All analyses were conducted using the R programming environment, version 4.1.0 (R Core Team, 2021).

We explored holdfast preference by seahorses (*i.e.,* a measure of the degree to which one habitat component is preferred over others; Curtis & Vincent, 2005) using Ivlev’s electivity index, calculated as follows: E = ri − ni/(ri +ni), where E is the measure of electivity, ri is the relative abundance (%) of seahorses using a certain type of holdfast, while ni is the relative abundance (%) of the same type of holdfast available in the micro-habitat. The index of electivity, or preference, varies from −1 to 1, where values between 0-1 indicate a preference and negative values indicate avoidance or random selection (Krebs, 1989).

## Results

**Figure 2:**
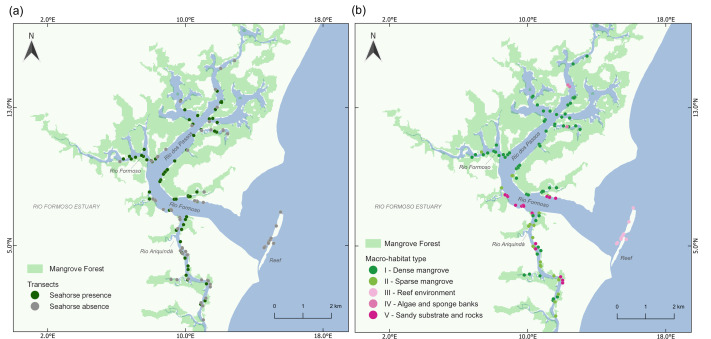
Map of the Rio Formoso Estuary (Pernambuco State, Brazil) showing (A) the presence or absence of seahorses at sampling sites, and (B) the type of macro-habitat at sampling points n each of the three rivers sampled (Rio dos Passos, Rio Formoso, and Rio Ariquindá), and on the reef (mouth of the estuary). The description of macro-habitat types can be found in Table 1.

**Figure 3:**
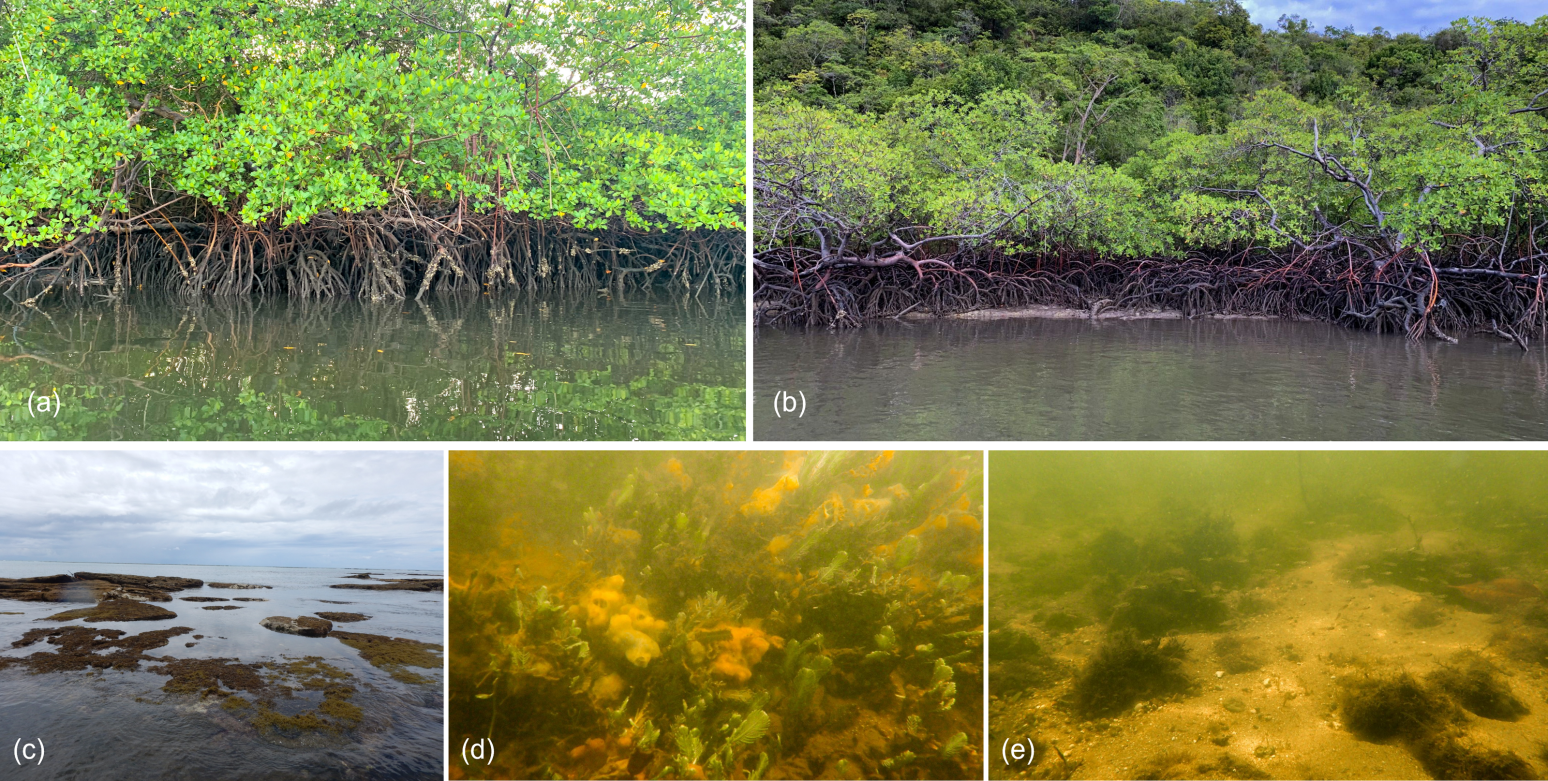
Macro-habitats types found in the sampling sites at the Rio Formoso Estuary (Pernambuco State, Brazil). (A) Dense mangrove, (B) sparse and/or tidal flat mangrove, (C) reef environment, (D) algae and sponge banks, and (E) sandy substrate with rocks. The description of macro-habitat types can be found in Table 1.

We found a total of 283 seahorses in the 125 transects, with 65 transects yielding one or more seahorses (Fig. 2A). We identified five categories of macro-habitat which are predominant in the transects sampled in the estuary: dense mangrove, sparse and/or tidal flat mangrove, reef environment, algae and sponge banks, and sandy substrate with rocks (Table 1; Fig. 3). Type I macro-habitat (dense mangrove) was recorded in most of the transects where at least one seahorse was sighted (74%; *n* = 48; Fig. 2B).

**Figure 4:**
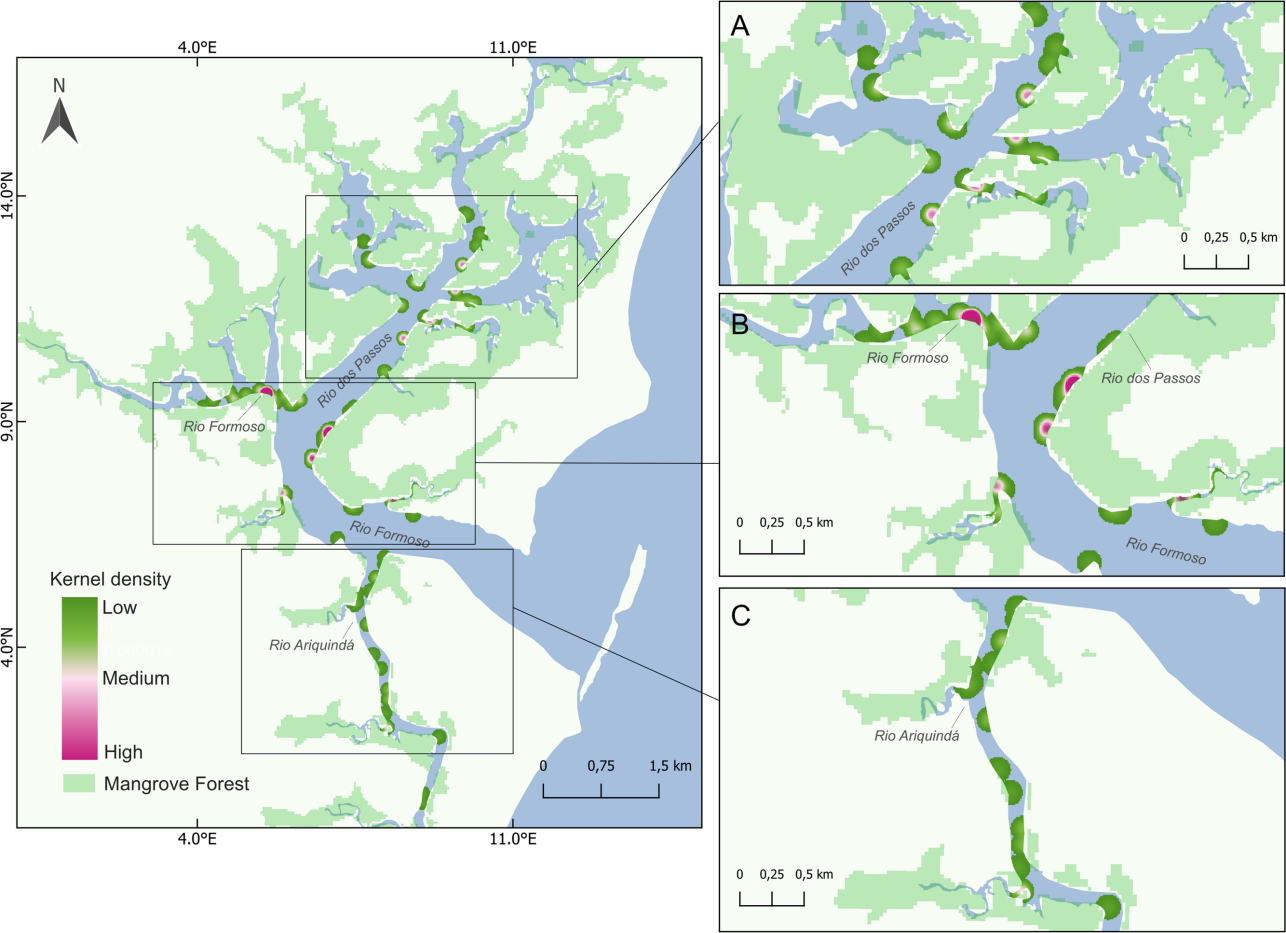
Map showing seahorse density hotspots at sampling sites in the Rio Formoso Estuary, with highlights for (A) Rio dos Passos, (B) Rio Formoso, and (C) Rio Ariquindá. The heat gradient represents areas with the highest density of seahorses by kernel density with a radius of 100 m.

Seahorse densities were unequally distributed across the estuary, ranging from 0 to 0.34 ind./m^2^ with a mean overall density of 0.022 ± 0.007 ind./m^2^. Highest seahorse densities were recorded in Rio Formoso (0.032 ± 0.023 ind./m^2^; Kruskal–Wallis test, *X*^2^ = 18.827; *df* = 3; *p* < 0.001; Fig. 4), followed by the Rio dos Passos (0.031 ± 0.011 ind./m^2^) and Rio Ariquindá (0.008 ± 0.004 ind./m^2^). Seahorse density was significantly correlated with the macro-habitat type (Kruskal–Wallis test, *X*^2^ = 26.448; *df* = 4; *p* < 0.001), the highest densities were recorded in transects with type I macro-habitat (dense mangrove; Table 1).

The optimal model indicated depth and habitat type as the explanatory variables predicting seahorse density in the RFE (*X*^2^ = 431.7; *df* = 117; Table 2). In contrast, the optimal model based on presence/absence data indicated only habitat type (*X*^2^ = 428.3; *df* = 117; Table 2). Both models were not overdispersed (*p* > 0.5). We found that the shallower the depth, the seahorse density increases. Regarding macro-habitat type, the increase of the presence of type V macro-habitat (sandy substrate with rocks) represents a decrease in density and in the probability of finding seahorses.

**Table 2:** Optimal models of environmental variables predicting seahorse presence/absence (i) and density (ii) in the Rio Formoso Estuary, Brazil.

**Model**	**Predictor variables**	**Estimate**	**Std. error**	**z value**	***p*** **-value**	**AIC**	**AICc**	Δ
i: Presence/absence ∼habitat + (1—river)						210.1	211	0
	Intercept	−0.398	0.142	−2.791	0.005 (^**^)			
	Habitat: II	−0.171	0.311	−0.551	0.581 (^ns^)			
	Habitat: III	−19.819	5789.562	−0.003	0.997 (^ns^)			
	Habitat: IV	−0.006	0.721	−0.010	0.992 (^ns^)			
	Habitat: V	−1.741	0.721	−2.414	0.015 (^*^)			
ii: Density ∼depth + habitat + (1—river)						447.7	449.0	0
	Intercept	1.586	0.354	4.476	7.61e−06 (^***^)			
	Depth	−0.014	0.006	−2.240	0.025 (^*^)			
	Habitat: II	−0.074	0.363	−0.205	0.837 (^ns^)			
	Habitat: III	−19.973	6991.369	−0.003	0.997(^ns^)			
	Habitat: IV	−0.449	0.729	−0.616	0.538 (^ns^)			
	Habitat: V	−2.217	0.733	−3.023	0.002 (^**^)			

**Notes.**

Significance levels were as followed: ns *p* > 0.05; * *p* ≤ 0.05; ** *p* ≤ 0.01; *** *p* ≤ 0.001.

**Figure 5:**
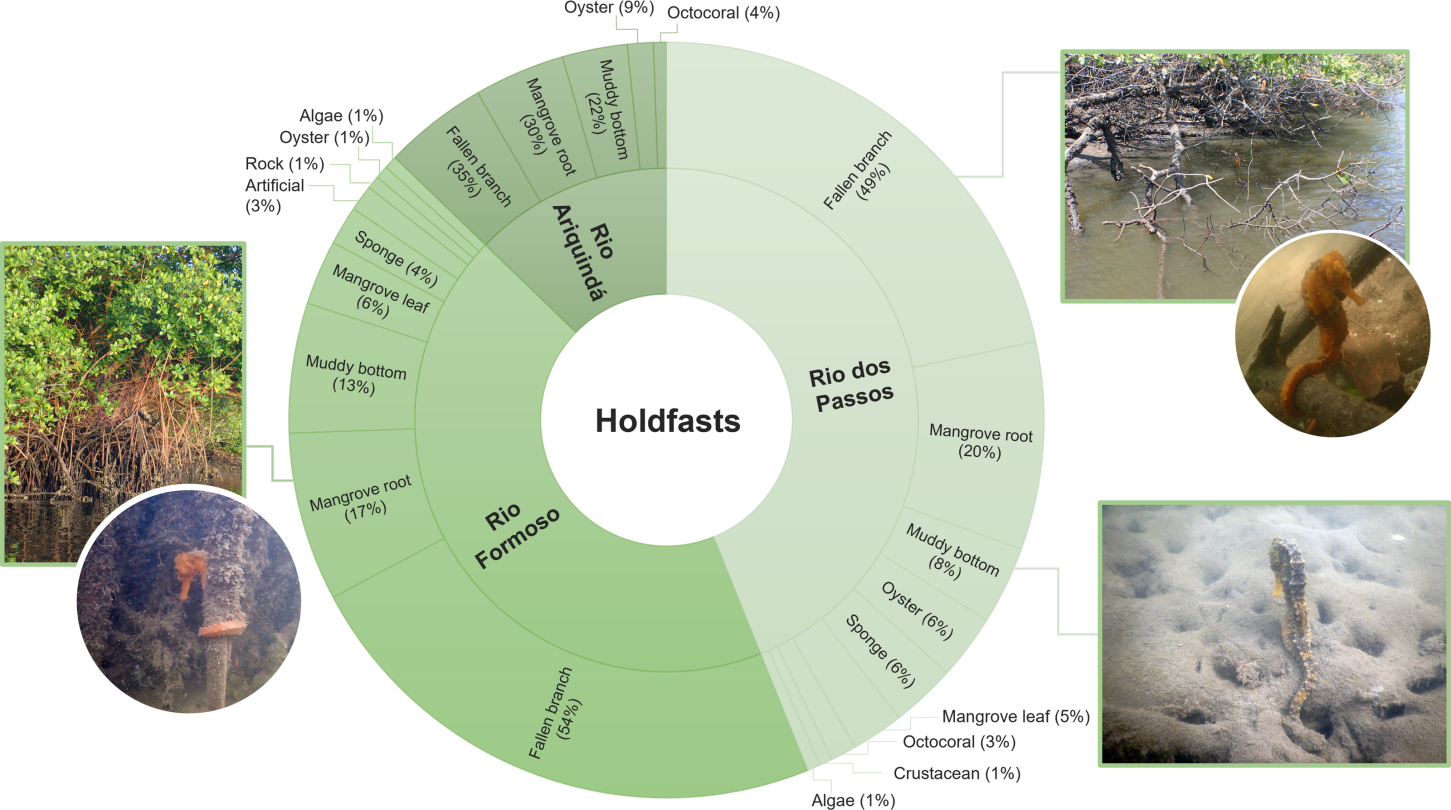
The proportion of holdfasts used by seahorses in each river sampled (Rio Formoso, Rio dos Passos, and Rio Ariquindá) at the Rio Formoso Estuary, Pernambuco State, Brazil.

Approximately 64% (*n* = 180) of all seahorses were recorded in stationary behaviour using 11 types of holdfasts (Table 3). The holdfast most used by seahorses were generally fallen mangrove branches (50%; *n* = 82), followed by mangrove roots (19.5%; *n* = 32) and muddy bottom (12.2%; *n* = 20). Fallen branches were also the most used holdfasts in the three rivers (Chi-squared test, *X*^2^ = 20.846; *df* = 20; *p* > 0.05; Fig. 5). According to the Ivlev’s electivity index, the seahorses preferred fallen mangrove branches as holdfasts, considering the entire estuary and each river specifically (Table 3).

**Table 3:** Ivlev’s electivity index for seahorse holdfast preference in the Rio Formoso Estuary, Brazil. The index is presented for the entire estuary (general) and its principal rivers. Values between 0-1 indicate a preference, and negative values indicate avoidance or a random selection. Values in bold indicate a preference.

**Holdfast**	**General**	**Rio dos Passos**	**Rio Ariquindá**	**Rio Formoso**
Algae	−0.88	−0.91	nr	−0.75
Leafy algae, articulated calcareous algae, red algae, filamentous green algae				
Artificial	−0.91	nr	nr	−0.80
Discarded tire, nylon rope				
Crustacean	−0.79	−0.56	nr	nr
Specimen of Paguroidea (shell)				
Fallen mangrove branches	**0.43**	**0.52**	**0.14**	**0.26**
Fallen mangrove leaves	−0.52	−0.21	nr	−0.55
Mangrove roots	−0.15	−0.11	−0.07	−0.19
From the species *Rhizophora mangle* and *Laguncularia racemosa*				
Muddy bottom	−0.75	−0.83	−0.64	−0.71
Octocoral	−0.71	−0.59	−0.39	nr
*Carijoa riisei*				
Oyster	−0.20	−0.08	**0.63**	nr
*Crassostrea* sp.				
Rock	−0.98	nr	nr	−0.95
Sponge	−0.81	−0.74	nr	−0.78
Unidentified species				

**Notes.**

nr not registered

## Discussion

This study contributes to a better understanding of the mechanisms driving seahorse distribution in a mangrove estuary. We found that depth and complex habitat types, primarily those formed by mangroves, are a key factor for *H. reidi*’s occurrence and density. In the RFE, the species was patchily distributed, as described in other studies in the area (Rosa et al., 2007; Oliveira, 2007). Moreover, it was found in low mean densities, as also reported in other mangrove estuaries in NE Brazil (between 0.006 ind./m^2^ and 0.51 ind./m^2^; Dias & Rosa, 2003; Rosa et al., 2007; Mai & Rosa, 2009; Schwarz Junior et al., 2021), following a general trend for seahorse populations (Lourie et al., 2004; Foster & Vincent, 2004). Different mechanisms might lead to seahorse patchy distribution patterns. Reproduction may be an important factor in explaining variations in seahorse movement and distribution, as has been demonstrated for *H. mohnikei*, which seems to undertake a seasonal inshore-offshore migration every year along the coast of China (Qin et al., 2017). Other mechanisms which potentially influence seahorse distribution are shelter and food availability (Curtis & Vincent, 2005) and predation (Kendrick & Hyndes, 2003; Manning et al., 2018), which are directly related to the habitat (Harasti, Martin-Smith & Gladstone, 2014).

Our two models (based on presence/absence and density) showed that macro-habitat type is a crucial factor for the distribution of *H. reidi* in the RFE, as we recorded the highest densities of *H. reidi* in type I macro-habitats (dense mangrove). In contrast, the presence of type V macro-habitats (sandy substrate and few rocks) reduces the probability of finding seahorses in the estuary. These results reflect a preference for more structurally complex habitats, possibly because these habitats provide abundant holdfast, shelter, and prey availability for seahorses (Curtis & Vincent, 2005; Harasti, Martin-Smith & Gladstone, 2014). Indeed, complex habitats play a direct role in seahorse distribution by supplying both the physical structure and indirectly the resources required for survival, growth, and reproduction (Manning et al., 2018). For instance, Curtis & Vincent (2005) reported that the distribution and abundance of *H. guttulatus* are influenced by the amount of habitat covered by vegetation and invertebrates, indicating a preference for more structurally complex habitats. Correia et al. (2018) also recorded preferences for specific types of habitats for *H. guttulatus* and *H. hippocampus*. Furthermore, it was demonstrated that the distribution of *H. capensis* in the Knysna Estuary (South Africa) is closely linked to suitable habitat (Teske et al., 2007). Our results reinforce the importance of this mechanism for the distribution of seahorses, showing that the distribution of *H. reidi* in a mangrove estuary is correlated with the type of macro-habitat, especially considering vegetation cover.

The availability of holdfasts has previously been pointed out by Dias & Rosa (2003) as a factor that positively influences the distribution of *H. reidi* in estuaries. In fact, we found *H. reidi* using different mangrove structures as holdfasts, such as roots, leaves, and fallen branches, showing a preference for the latter. The importance of habitats for seahorses was also demonstrated by Correia et al. (2015), who showed that fluctuations in *H. guttulatus* populations in Lagoa da Ria Formosa, Portugal, were positively correlated with the availability of holdfasts. Moreover, Harasti (2016) showed that a significant decline in the abundance of *H. whitei* in Australia was related to the reduced availability of marine habitats for the species. The preference of seahorses for habitats where they can hold on with their prehensile tail is fundamentally related to survival. As they are poor swimmers and inhabit shallow waters, this behavior allows them to protect themselves against predation and different environmental variables, such as currents (Lourie, Vincent & Hall, 1999; Claassens & Hodgson, 2018). Furthermore, as seahorses are considered sit-and-wait predators and rely heavily on crypsis, the availability of holdfasts is important for their feeding (Lourie, Vincent & Hall, 1999; Foster & Vincent, 2004). For example, habitat, prey, and predator variables were all significant correlates with *H. whitei* abundance in an estuarine environment, providing evidence that seahorses selected more complex habitats because it improved their success as ambush predators (Manning et al., 2018).

Our models also showed that *H. reidi* density in the estuary is positively related to shallower depths (<1 m). Similarly, Aylesworth et al. (2015) revealed that depth was one of the most critical environmental characteristics in predicting the presence of seahorses in mangrove estuaries in NE Brazil. Recognizing the existence of this pattern emphasizes the importance of coastal habitats for the *H. reidi*, especially mangroves, as well as for other seahorse species (Foster & Vincent, 2004; Rosa et al., 2007). The longsnout seahorse has a coastal distribution pattern (Rosa et al., 2007), being typically found at depths between 0.1 and 55 m (Vari, 1982; Rosa, Dias & Baum, 2002), but in mangrove estuaries in NE Brazil, average depths tend to be the lowest for the species (Rosa et al., 2007). This can be related to the fact that mangrove forests and the structures provided by them (*i.e.,* roots, branches) are present in the shallowest parts of the rivers (*i.e.,* river banks, tidal flats), emphasizing the relevance of this habitat availability for the occurrence of *H. reidi*, as has also been demonstrated by other studies (Rosa et al., 2007; Aylesworth et al., 2015; Xavier, 2009).

Seahorse density was significantly lower in the Ariquindá river. It is important to highlight that this river has been under high pressure of heavy boat traffic for decades (Santos, 2002; Rosa et al., 2007; Selva, 2012; Araújo, Alves & Simões, 2014), where there is previous evidence of direct implications of the boat traffic affecting the behavior of seahorses (Bruto-Costa, 2007). Indeed, the noise produced by heavy boat traffic has been suggested to promote behaviour changes in *H. capensis* in an estuarine environment (Claassens & Hodgson, 2017). The negative effects of boat traffic on fish populations can be attributed to noise, pollution, and the physical effects of boat movement (Whitfield & Becker, 2014), which can be potentialized in shallow estuarine systems due to their spatial limitations in terms of depth and width, especially during the low tide periods (Becker et al., 2013). Therefore, seahorse distribution and density in the RFE might have been affected by such anthropogenic pressures, which deservers further investigation.

Our findings show that mangroves are crucial habitats for *H. reidi* and we highlight that conservation measures towards the species must consider mangrove protection. Shallow marine habitats are ecologically and socio-economically important as these habitats provide natural resources and ecosystem services crucial for human survival and well-being (Magris & Barreto, 2010; Pelage et al., 2019). But still, they are often the most heavily degraded by anthropogenic activities. Mangroves are one of the most threatened ecosystems in the world due to human pressure (Alongi, 2002), with estimates that at least a third of mangrove forests have been lost globally in the last 60 years (Hamilton & Casey, 2016; Pelage et al., 2019). Although mangroves are protected by law throughout the national territory in Brazil, and ca. 80% of its coverage is inserted in protected areas, more than 25% have been deforested in recent years (Fundação SOS Mata Atlântica, 2020). Most impacts are due to an increase of activities related to agriculture, industry, tourism, and shrimp farming (Schaeffer-Novelli et al., 2000; Guimarães et al., 2010). Moreover, recent Brazilian political decisions, such as changes in the Mangrove National Action Plan (PAN Manguezal) with the removal of the important objective of eradicating shrimp farming and saline developments in mangrove systems, left Brazilian mangrove ecosystems even more unprotected and vulnerable (Ottoni et al., 2021).

Protecting mangroves for protecting seahorses can also be a two-way street. Seahorses are charismatic, especially emblematic animals that are considered flagship species for conservation issues (Rosa, Dias & Baum, 2002; Vincent, Foster & Koldewey, 2011). In this sense, they can provide a powerful opportunity to garner considerable political and public support for conservation strategies in mangroves, estuaries, and other shallow marine habitats. It has been demonstrated that designating marine protected areas for estuarine seagrass habitats based on syngnathids density and assemblage variation may benefit other fish species (Shokri, Gladstone & Jelbart, 2009). This potential could be pivotal for promoting the conservation of mangroves. As threatened species, the conservation goals for seahorses include the protection of habitats that are important to them, so, their association with mangroves can foster the conservation of this ecosystem vital to the survival of *H. reidi* and other coastal and marine species. It is important to notice that although *H. reidi* is a nationally threatened seahorse species, which primarily occurs in mangroves in NE Brazil (Rosa et al., 2007; Dias-Neto, 2011), it has not been considered a focal species in the ‘PAN Manguezal’ (Ministry of Environment and Climate Change of Brazil (MMA), 2019). This requires more attention to strengthen the mechanisms for concomitantly protecting seahorses and mangroves in the country, especially considering the flagship potential of these animals.

## Conclusions

This study showed that habitat type and depth are the main factors influencing the distribution of *H. reidi* in a mangrove estuary. The species’ preference for habitats formed by mangroves and the holdfasts that mangrove structures provide is clear. Knowing that these habitat characteristics are key factors for the species, it is now necessary to assess the current protection of estuaries with mangroves where seahorses are known to occur and identify areas that should be included in future protected areas as a priority. Monitoring seahorse populations in these environments is equally necessary to identify possible fluctuations and their causes. Engaging local stakeholders as decision-makers (*e.g.*, local communities, authorities, and the local tourism sector) is crucial for any future action to be effective. Integrated with knowledge about the distribution of seahorses in estuaries, these efforts can provide the necessary tools to guide existing strategies and develop new conservation measures to ensure the protection that seahorses require as threatened species.

##  Supplemental Information

10.7717/peerj.15730/supp-1 Data S1 Raw data

## Additional Information and Declarations

### Field Study Permissions

The following information was supplied relating to field study approvals (*i.e.*, approving body and any reference numbers): Permission was approved by Sistema de Autorização e Informação em Biodiversidade - SISBIO (license no. 59294-2)

### Data Availability

The following information was supplied regarding data availability: The raw data shows the variables used in the tests (depth, salinity, temperature, macro-habitat type) to verify seahorses’ habitat preferences and is available in the Supplemental File.
